# Classification of fracture and non-fracture groups by analysis of coherent X-ray scatter

**DOI:** 10.1038/srep29011

**Published:** 2016-07-01

**Authors:** A. J. Dicken, J. P. O. Evans, K. D. Rogers, N. Stone, C. Greenwood, S. X. Godber, J. G. Clement, I. D. Lyburn, R. M. Martin, P. Zioupos

**Affiliations:** 1 Imaging Science Group, Rosalind Franklin Building, Nottingham Trent University, Nottingham, UK; 2 Cranfield Forensic Institute, Cranfield University, Shrivenham, UK; 3 Biomedical Physics Group, Physics and Astronomy, University of Exeter, Exeter, UK; 4 Halo X-ray Technologies, MediCity, Nottingham, UK; 5 Melbourne Dental School, University of Melbourne, Melbourne University, Melbourne, Australia; 6 Medical Imaging Department, Cobalt Health, Cheltenham, UK; 7 Epidemiology and Public Health, School of Social and Community Medicine, University of Bristol, Bristol, UK

## Abstract

Osteoporotic fractures present a significant social and economic burden, which is set to rise commensurately with the aging population. Greater understanding of the physicochemical differences between osteoporotic and normal conditions will facilitate the development of diagnostic technologies with increased performance and treatments with increased efficacy. Using coherent X-ray scattering we have evaluated a population of 108 *ex vivo* human bone samples comprised of non-fracture and fracture groups. Principal component fed linear discriminant analysis was used to develop a classification model to discern each condition resulting in a sensitivity and specificity of 93% and 91%, respectively. Evaluating the coherent X-ray scatter differences from each condition supports the hypothesis that a causal physicochemical change has occurred in the fracture group. This work is a critical step along the path towards developing an *in vivo* diagnostic tool for fracture risk prediction.

There is a growing need to understand and quantify the condition of osteoporotic and normal tissues at the atomic level and the concomitant differences at the physicochemical level. This information is required to support and inform the development of accurate diagnostics capable of enhanced discrimination. Osteoporosis is a systemic skeletal condition characterised by low bone mass and the micro-architectural deterioration of bone tissue, with a consequent increase in bone fragility and susceptibility to fracture^1^. The increased fracture risk due to osteoporosis has a considerable impact on morbidity and mortality; it is a major health economic issue. It is estimated that one in three women over fifty years old will experience osteoporotic fractures, as will one in five men^2^. Even marginal improvements in diagnostic performance would be of very significant benefit^3^. Whilst osteoporosis related fractures can occur at any time their probability appears to increase exponentially with age^4^. Given the aging population it was estimated in 2011 that by 2025, annual direct costs from osteoporosis in the US alone are expected to reach approximately $25.3 billion^5^.

The diagnosis of osteoporosis is based upon the assessment of bone mass and quality^6^. However, Kanis (2002) argues that there are no satisfactory clinical means to assess bone quality and therefore diagnosis of osteoporosis depends upon the measurement of skeletal mass. Currently, in the clinical setting, skeletal mass is estimated by dual-energy X-ray absorptiometry (DEXA) which enables the calculation of bone mineral density (BMD). Bone strength and thus fracture risk are correlated with BMD although only ~70% of the variation in compressive bone strength can be attributed to mineral density alone^7^. It therefore follows that establishing and measuring the factors attributed to the remaining ~30% would lead to the development of improved patient management and a reduced health burden. There is support in the literature that physicochemical properties of bone mineral (referred to as bone quality^8^) are also required for accurately predicting bone strength^9^,^10^,^11^,^12^,^13^,^14^,^15^, though the precise relationships between these properties remain elusive. The measurement of coherent X-ray scatter or diffraction, which for brevity is termed X-ray scatter in the remaining text, may be employed to calculate several factors associated with bone quality^9^ and more recently has shown potential for operation at diagnostically appropriate X-ray energies^16^,^17^. These measurements are independent of conventional bone mineral density (DEXA scans) that provides estimates of the average areal density of all bone components. The scatter signatures arise almost entirely from the apatite mineral’s crystallographic periodicity and therefore may be considered orthogonal to BMD.

We have collected X-ray scatter patterns from 108 bone samples (54 from individuals suffering from hip fractures and 54 from individuals with no fractures) and used this information in two different ways. First, to build a classification model that predicts each condition to produce a fracture and non-fracture group; and second, to evaluate which characteristics within the scatter patterns may be condition related. This approach was designed to further our understanding of the physicochemical changes that occur in osteoporotic tissue. Ultimately, our aim is to support and inform the ongoing development of an *in vivo* diagnostic technique to enhance fracture risk prediction.

## Results

**Figure 1:**
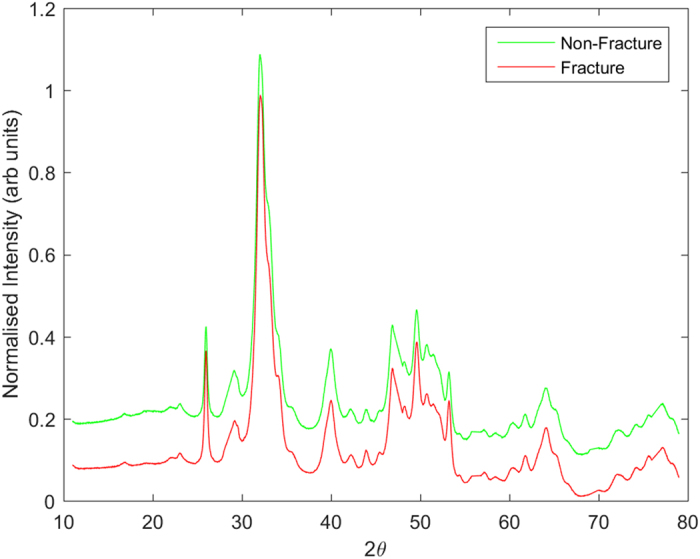
Comparison of min-max normalised mean diffractograms from fracture and non-fracture bone samples, where 2θ is the angle subtended by the trajectory of the diffracted or scattered X-rays with respect to the interrogating beam. The plots are offset along the vertical axis for clarity.

The fracture group comprised of 54 samples from 19 patients. The non-fracture group comprised 54 samples from 54 individuals. A total number of 108 diffractograms were recorded; one for each sample. The min-max normalised mean diffractograms from each condition are illustrated in Fig. 1.

**Figure 2:**
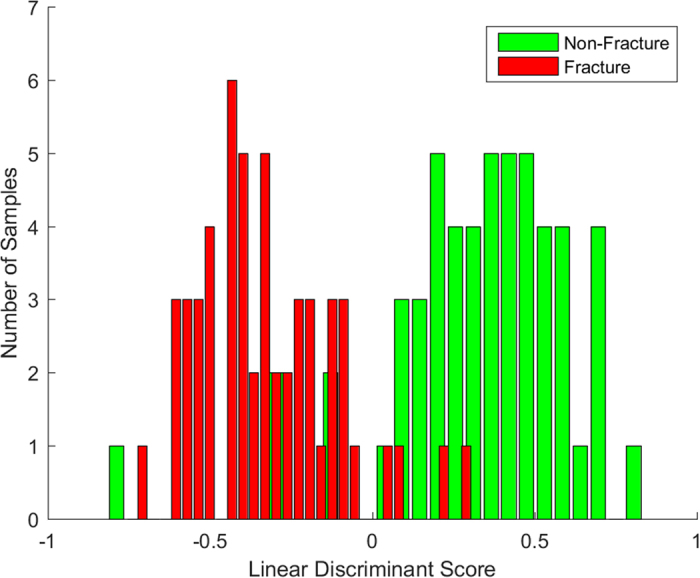
Two group histogram demonstrating classification performance of the two group model; non-fracture versus fracture.

A 2-group principal component fed linear discriminant classification model was developed and applied to separate the fracture and non-fracture groups. Leave one sample out cross-validation demonstrated a sensitivity of 93% and a specificity of 91%. Figure 2 illustrates a histogram of linear discriminant scores for each diffractogram (colour codes for fracture and non-fracture groups), which demonstrates the potential diagnostic capability of X-ray scattering to separate these groups.

**Figure 3:**
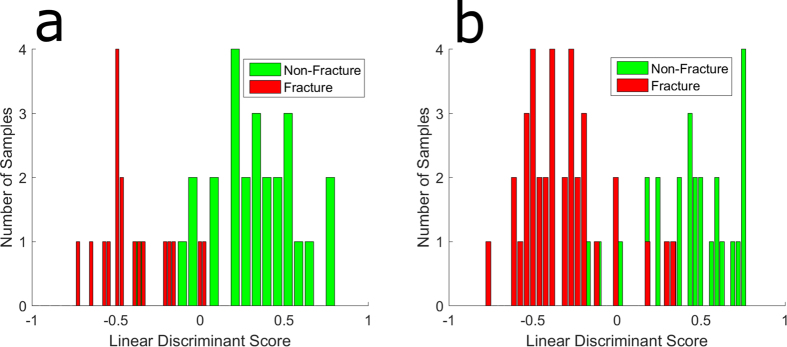
Two group histogram demonstrating classification performance of the two group model; non-fracture versus fracture for males (**a**) **and females** (**b**).

This method was repeated on a subset of data to evaluate this approach for discriminating fracture and non-fracture groups between the sexes. Classification resulted in 89% sensitivity and 89% specificity for males; 92% sensitivity and 89% specificity for females. Histograms of the linear discriminant scores for each sex are illustrated in Fig. 3.

In addition, our analysis was modified to provide an independent validation of this approach. We applied a leave one patient out cross-validation in which no samples from the test patient were included in the training model. The resultant sensitivity of 93% and a specificity of 91% were unchanged in comparison with the leave one sample out cross-validation method.

It is unlikely that an XRD technique capable of making *in vivo* measurements^16^,^17^ would be able to achieve the measurement fidelity provided by the conventional laboratory diffractometer employed in this study. To explore the implications likely of an *in vivo* system the classification analysis was repeated after re-interpolating the raw data with increased step sizes, see Table 1.

**Figure 4:**
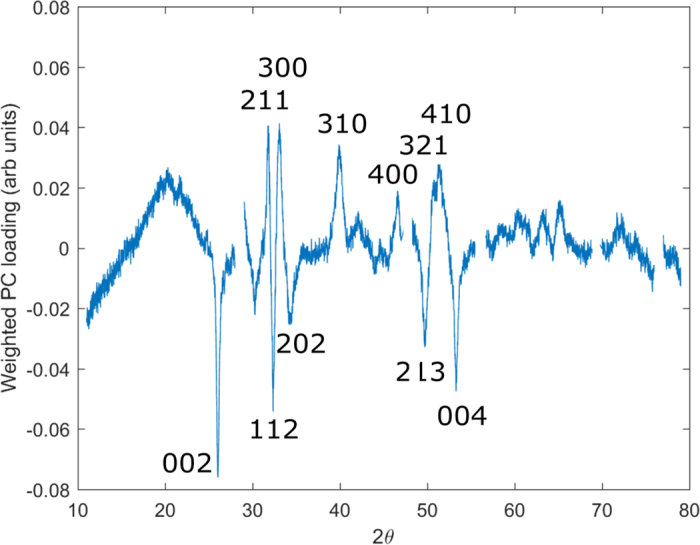
Sum of principal component loadings weighted by their significance at classifying fracture and non-fracture groups (using those weightings calculated by the linear discriminant analysis (LDA) model).

Further analysis was undertaken to evaluate the differences in the scatter patterns and thus by inference, the potential physicochemical differences upon which the classification was based. Principal component loadings e.g. analogous to correlation coefficients, provided the variable (in this case 2θ) explained by each component. Linear discriminant weights generated after linear discriminant analysis identified three principal components as being responsible significantly for the classification (p < 0.05). A weighted sum of the associated PC loadings and thus a weighted sum of the variation in the scatter angle attributed to classification is illustrated in Fig. 4. In this circumstance negative peaks are indicative of the fracture group and positive peaks are indicative of the non-fracture group.

A number of the prominent peaks that appear important for discriminating between the groups are labelled with their crystallographic Miller indices.

## Discussion

The performance of DEXA is at least as good at diagnosing osteoporosis as blood pressure is at predicting a stroke^6^, though consistent performance statistics are not available in the literature. Enhancements have already been introduced in the form of patient risk factors but we seek to include additional information from bone quality that cannot be measured by bone mineral density (BMD).

The measurement of scattered X-rays from bone enables specific material characteristics of the mineral content to be determined. We recorded diffractograms from 108 bone samples *ex vivo* relating to fracture and non-fracture groups. Principal component fed linear discriminant analysis was selected as a multivariate approach to develop a classification model to distinguish between each group and by hypothesis each condition. This method was adopted as it provides specific information on the variance in the dataset and the relative importance of features in the diffractogram, i.e. it is not a black box method. These key components were then used to explore whether they could be used to discriminate conditions based upon maximising the between group variance and minimising the within group variance with kindest discrimination analysis. Classification performance was found to be extremely high, for this dataset, yielding a sensitivity and specificity of 93% and 91%, respectively. Repeating this analysis for each sex resulted in slightly better performance for female classification (92% sensitivity and 89% specificity) in comparison to male classification (89% sensitivity and 89% specificity), which is perhaps attributable to lower sample numbers for training. Importantly, this performance appears to exceed significantly that expected from DEXA in a clinical setting. Also, in these circumstances XRD and DEXA would provide independent assessments of fracture risk. Our conjecture is that by combining data from each technique it may be possible to improve significantly the overall performance of diagnostic testing. Techniques that have the potential to record X-ray scatter patterns *in vivo* are in their infancy. However, it is unlikely that even a more mature implementation applied to the femur would achieve the measurement fidelity provided by a laboratory diffractometer operating under ideal conditions. Emulating a reduction in measurement fidelity, see Table 1, did not reduce significantly the classification performance. This is a promising result with regard our planned *in vivo* studies and an important step along the path to the implementation of an *in vivo* instrument.

Understanding the signal characteristics that underpin the classification and thus identify possible physicochemical changes between conditions is paramount as it could lead to the development of treatments with increased efficacy. Figure 4 illustrates the peaks in the coherently scattered X-ray intensity that contributes most significantly to the classification. In general, the quantity of scattering from the non-fracture group was greater than that from the fracture group. This observation is attributed to a systemic difference in the mass of sample analysed i.e. the slightly different mechanical sampling regimes employed produced relatively more non-fracture sample mass. The scatter data demonstrates a broad intensity peak at ~20°/2θ that is enhanced in the non-fracture group. The collagen helical rise per residue is ~0.29 nm and produces a broad peak at ~30°/2θ. Therefore a difference plot characteristic is more likely attributable to variations in the overall lipid content of the samples. Small methodological differences in the fat removal stage of the sample preparation may therefore account for this observation. Thus a subset of the scatter patterns with this peak excluded was evaluated. The performance of the classification was not affected significantly i.e. sensitivity and specificity of 89% and 85%, respectively, indicating that successful classification is not dependent upon this feature.

Although bone performs biologically critical mechanical and homeostatic functions, the relationships between its hierarchical constituents are not well understood. The basis chemical composition (non-stoichiometric hydroxyapatite) crystallises into a variable ultra-structure (nano-crystallites) that together with organic components form the fundamental building blocks of bone’s microarchitecture. Coherent X-ray scatter provides information specifically regarding the physicochemical characteristics of bone mineral. There is significant evidence from previous studies that bone mechanical properties (e.g. fragility) are affected by these physicochemical features^9^, although there remains controversy concerning the precise nature and magnitude of such relationships^18^. The composition of apatite is known to markedly affect its crystallite size and shape. For example, phosphate substitution by carbonate (bone apatite contains ~5%wt CO_3_^2−^) results in smaller, more rod like crystallites than the corresponding unsubstituted chemistry^19^ as the increase in lattice disorder produces increased solubility of the crystallites^20^. This type of substitution must be accompanied by heteroionic exchange or vacancies at the anionic calcium sites for charge balance.

Within this study, there are a number of differences associated clearly with the Bragg maxima arising specifically from the nano-crystalline, mineral apatite. In general, such differences can arise from systematic shifts in peak positions and/or intensity changes (both indicative of apatite unit cell modification) and peak shape variation (suggestive of microstructural revisions). Unfortunately, X-ray diffraction from nano-crystalline materials with relatively low symmetry, such as biological apatite, is characterised by significant Bragg peak overlapping and thus equivocally associating features of difference plots with specific structural differences is problematic. For example, features within Fig. 4 at scatter angles between 30–35°/2θ are consistent with both microstructural and cell dimension differences between the groups although both are indicative of lattice substitutions. Reduced crystallite size of bone mineral has previously been associated with decreased load accommodation and increased fracture risk^21^ and is observed consistently in pathologies such as osteoporosis imperfecta^22^. It has also been proposed that an ‘optimal’ crystallite size corresponds to maximum bone strength^10^.

Our results also show that the Bragg peak intensity differences are related to specific crystallographic directions; planes normal to the basal plane produce increased intensity within the non-fracture group. This aspect may be related to systemic differences within the unit cell chemistry. For example, the effect of reducing the Ca^2+^ occupancy (or electron density for those ions at the channel sites) is to increase the structure factor of the 002 and 004 reflections and decrease the corresponding amplitude of scatter for the 310. This is entirely consistent with the observations of this study and indicates possible specific differences in apatite chemistry between the fracture and non-fracture groups. Disorder within the apatite lattice, as introduced by such lattice site modifications, has been proposed as a fundamental contributor to bone mechanical compromise for more than three decades^23^ and evidence for this hypothesis continues to be reported^24^. Consequently, the chemical composition of bone apatite through its influence on crystallite dimensions and subsequently micro-architecture is a significant determinant of bone mechanical performance. Thus these features may influence coherent scatter measurements to distinguish between bones with different fragilities.

## Methods

### Bone Samples

A total of 19 femoral heads, from individuals who had suffered trauma fractures at the femoral neck, were donated by patients after they had undergone hip replacement surgery. From these, 54 samples were taken randomly across the femoral head to comprise our fracture group. Ethical approval was provided by the Gloucestershire NHS Local Research Ethics Committee. The non-fracture group derived from the femoral heads of 54 individuals donated from the Melbourne Femur Collection. Informed consent was obtained from the next of kin in strict accordance with Australian National Health and Medical Research Council guidelines and prevailing local legislation, and ethics approval provided by the University of Melbourne. All methods in handling the material for the work reported in this paper were carried out in accordance with the approved guidelines and the appropriate standards applying in this medico-legal context. Further details are provided in the Acknowledgements.

The Australian and UK donors were all taken from an Anglo-Celtic population with a common Western lifestyle. A demographic break down of the samples is illustrated in Table 2.

### Sample Preparation

The precise cleaning procedure for the fracture group samples has been detailed elsewhere^25^, but in summary each sample was subject to a high pressure jet wash and soaked in 1:1 mix of chloroform and methanol to remove fat. All bone samples were homogenised using a Retsch mixer miller (mm 2000) with zirconium milling baskets and balls. Each sample was sectioned (to reduce milling time) and milled in 60 second cycles with a 60 second rest period between cycles to prevent overheating (which is known to affect crystallinity^26^). The samples were then sieved through a 106 μm stainless steel mesh to ensure homogeneity.

### X-ray Scattering

The angular distribution of the X-ray scatter was measured using conventional X-ray diffraction (XRD) methods. Samples were loaded into holders made from low-background off-cut silicon. To correct for differences in sample position, each sample was spiked with a small amount of silicon powder (NIST SRM640c). Systematic peak shifts due to sample position were corrected as a post processing step and re-interpolated onto a common scatter angle (2θ) scale.

The XRD analysis was conducted by a PANalytical X’pert Pro Diffractometer with Cu target operating at 40 keV and 40 mA. A PIXcel strip detector collected scattered photons from 10–80° 2θ in 0.013° steps with an equivalent integration time of 150 seconds per point.

### Statistical Analysis

Silicon peaks were removed from each diffractogram as a pre-processing step to ensure that any small differences in the levels of dopant would not bias classification. Each diffractogram was normalised and mean centred by subtracting the mean of all diffractograms from each individual diffractogram. Principal component fed linear discriminant analysis was employed to develop a classification model in Matlab®. Principal components (PCs) were generated that simplified the data while retaining the salient information required for classification. Each PC was tested with analysis of variance (ANOVA) and PCs with a high significance (p < 0.05) for classification were selected for inclusion in the linear discriminant analysis (LDA) models. Leave-one-out cross-validation and linear discriminant analysis was used to calculate sensitivity and specificity^27^. This analysis involved the exclusion of one sample at a time and a full recalculation of the mean-centring, PCs and ANOVA selection of significant PCs, prior to projection of the ‘independent’ sample onto the model for prediction of fracture/non-fracture group. This process was repeated for each sample.

## Additional Information

**How to cite this article**: Dicken, A. J. *et al*. Classification of fracture and non-fracture groups by analysis of coherent X-ray scatter. *Sci. Rep*. **6**, 29011; doi: 10.1038/srep29011 (2016).

## Figures and Tables

**Table 1:** Classification performance of identifying fracture and non-fracture groups from X-ray scatter patterns when re-interpolated at increasing step sizes.

Step Size (^o^)	Sensitivity (%)	Specificity (%)
0.013	93	91
0.1	93	91
1	89	87
2	87	85

**Table 2:** *Ex vivo* bone sample demographic break down.

	Fracture		Non-Fracture	
	Males	Females	Males	Females
Samples	18	36	27	27
Donors	4	15	27	27
Age range	74–78	73–90	66–93	60–90
